# Investigation of chain-length selection by the tenellin iterative highly-reducing polyketide synthase†

**DOI:** 10.1039/d3ra08463a

**Published:** 2024-03-15

**Authors:** Katharina Schmidt, Russell J. Cox

**Affiliations:** a OCI, BMWZ, Leibniz University of Hannover Schneiderberg 38 30167 Hannover Germany russell.cox@oci.uni-hannover.de

## Abstract

The programming of widely distributed iterative fungal hr-PKS is mysterious, yet it is central for generating polyketide natural product diversity by controlling the chain length, β-processing level and methylation patterns of fungal polyketides. For the iterative hr-PKS TENS, responsible for producing the pentaketide–tyrosine hybrid pretenellin A 1, the chain length programming is known to be determined by the KR domain. Structure prediction of the KR domain enabled the identification of a relevant substrate binding helix, which was the focus of swap experiments with corresponding sequences from the related hr-PKS DMBS and MILS that produce similar hexa- and heptaketides (2, 3). The investigations of chimeric TENS variants expressed *in vivo* in the host *Aspergillus oryzae* NSAR1 revealed the substrate binding helix as a promising target for further investigations, evidenced by observed increase of the chain length during swap experiments. Building on these findings, rational engineering of TENS was applied based on structural analysis and sequence alignment. A minimal set of four simultaneous amino acid mutations achieved the re-programming of TENS by producing hexaketides in minor amounts. To refine our understanding and minimize the number of mutations impacting polyketide chain length, we conducted an alanine scan, pinpointing crucial amino acid positions. Our findings give indications on the intrinsic programming of hr-PKS domains by minimal changes in the amino acid sequence as one influence factor for programming.

## Abstract 2

Engineering the substrate-binding-helix of the keto-reductase domain of TENS controls chain-length selectivity of the products.

## Graphical abstract

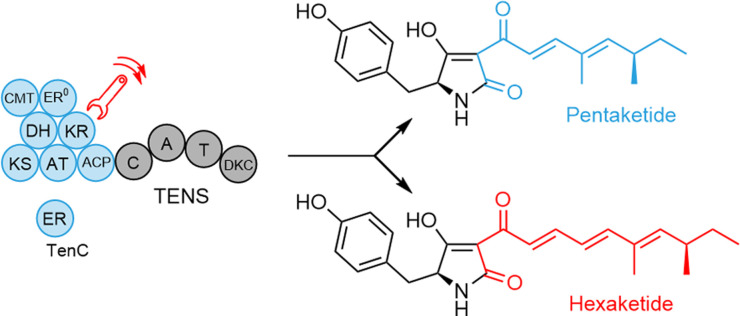

## Introduction

Fungal iterative highly-reducing polyketide synthases (hr-PKS) are multidomain enzymes that are closely related to vertebrate fatty acid synthases (vFAS).^1^ These systems consist of several covalently linked catalytic domains that form a dimeric megasynthase.^2^ In some cases crystallography (*e.g.* vFAS)^1,3^ and cryogenic electron microscopy (cryo-EM, *e.g.* hr-PKS)^4^ have given structural details for these enzymes, but in the vast majority of cases structural data is not available. However, significant progress has been made in applying biochemical methods that can illuminate aspects of selectivity and programming, particularly in the case of the hr-PKS.

vFAS and hr-PKS enzymes are iterative systems that use an acyl-CoA starter unit (usually acetyl) that is loaded onto the ketoacylsynthase domain (KS). An acyl transferase (AT) domain then loads a malonyl group from malonyl CoA onto the phosphopantetheine (PP) prosthetic group of the acyl carrier protein (ACP) domain. The KS then catalyses a decarboxylative Claisen reaction to form acetoacetyl ACP. In vFAS a cycle of β-processing reactions then reduces the ketone to an alcohol (ketoreductase, KR) using NADPH, eliminates the β-alcohol (dehydratase, DH), and finally reduces the enoyl system (enoyl reductase, ER) with NADPH to form butyryl ACP. This can enter another round of extension and β-processing, and the iterative cycle continues in vFAS until the fully saturated chain reaches C_16_ or C_18_ when it is hydrolytically off-loaded by a thiolesterase (TE) domain.^5,6^

hr-PKS enzymes catalyse the same extension reactions, but an additional reaction includes methylation at the α-carbon by *S*-adenosyl methionine (SAM) catalysed by a *C*-methyltransferase (*C*-MeT) domain. hr-PKS also differ because the β-processing reactions and eventual chain-length of the polyketide are highly programmed so that specific, but highly varied compounds can be synthesised by different hr-PKS.^7^ Understanding and engineering the programme of hr-PKS is a major focus of research.^7^

**Scheme 1:**
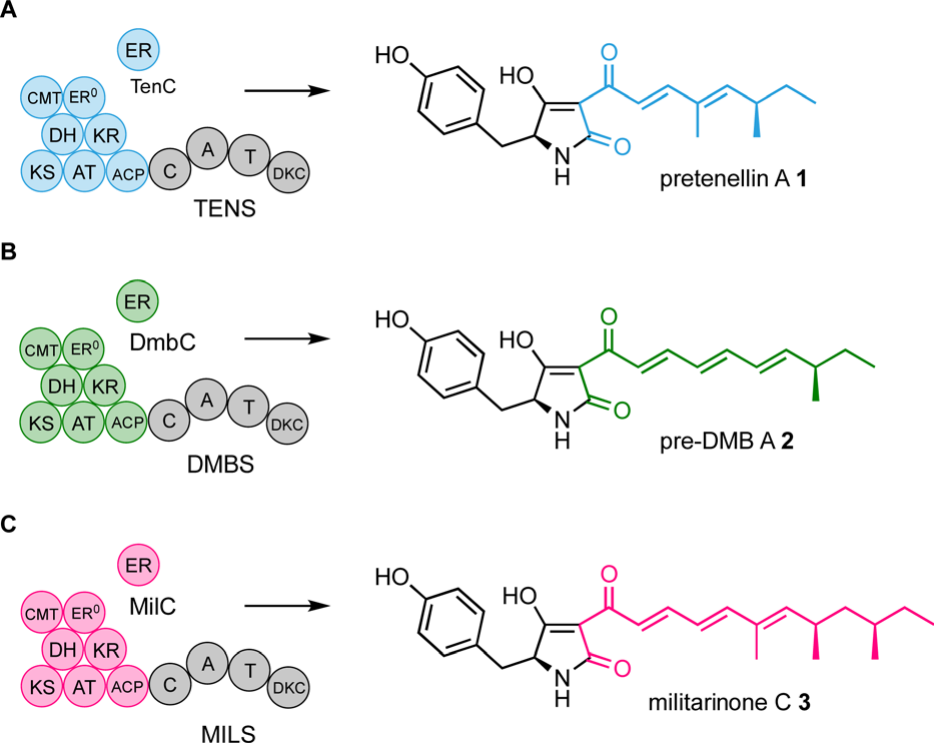
Observed functions of the programmed PKS-NRPS systems TENS, DMBS and MILS. (A) TENS + TenC produces pentaketides; (B) DMBS + DmbC produces hexaketides; (C) MILS + MilC produces heptaketides.

We have focussed efforts on the pretenellin A 1 hr-PKS TENS. This is a hybrid system where the off-loading is achieved by a non-ribosomal peptide synthetase (NRPS) that consists of condensation (C), adenylation (A), thiolation (T), and Dieckmann cyclase (DKC) domains (Scheme 1A).^8^ TENS builds a doubly methylated pentaketide that is attached at the hr-PKS ACP. The TENS hr-PKS has an inactive ER domain (ER^0^), but a *trans*-acting ER (TENC) fulfils the catalytic role. TENC also plays a part in enforcing the fidelity of the hr-PKS programme.^9^ When the pentaketide intermediate cannot be extended, it is passed to the NRPS where it is linked to tyrosine and cyclised and released as pretenellin A 1 (Scheme 1A).^8,10,11^

TENS belongs to a large family of related hr-PKS-NRPS systems that can synthesise different related polyketides.^12^ For example the desmethylbassianin synthetase (DMBS) produces the hexaketide predesmethyl bassianin A 2 (preDMB A, Scheme 1B), while the militarinone synthase (MILS) produces the heptaketide militarinone C 3 (Scheme 1C).^8,9,13^ These hr-PKS systems are very similar at sequence level, but differ in their programmes of methylation and chain length determination.^12^

Our approach to study the mechanism of programming of these hr-PKS has been to perform domain swaps, using TENS as the acceptor and DMBS and MILS as donors.^9,12,14,15^ In early experiments we showed that removal of the TENS KR and its replacement with the DMBS KR resulted in the production of new hexaketides – *i.e.* an *increase* in chain length.^15^ Later experiments swapped much shorter fragments of the KR and showed that exchange of a 69-residue region was sufficient to show chain-length changes. The same 69-region swap from the MILS produced heptaketides.^12^ Finally, swap of a 12-residue sub-region from MILS that corresponds to a helix in the KR active site also resulted in the production of heptaketides.^12^

The observation that the KR domain affects chain-length was interpreted as indication of kinetic competition between catalytic domains.^12^ Since the KS does not appear to extend β-ketones, lack of KR activity commits polyketide intermediates to off-loading by the NRPS. Thus, the selectivity of the KR either commits intermediates to further processing and chain-extension when it is active, or to off-loading when it is not.^12^ Here, we aimed to investigate the selectivity of the TENS KR domain in more detail by focussing our efforts on an α-helix adjacent to the active site, which was speculated to be in contact with the bound substrate during the reduction.

## Results

### Model building

There are no existing X-ray or cryo-EM structures of the TENS hr-PKS or of the individual KR domain. However, there are structures of vFAS that have been used previously as models, and there are known structures of KR domains from modular bacterial PKS that have served as templates for the development of threaded models of the TENS KR domain in the past.^12^ It is known that the TENS KR is a B-type KR, indicated by the characteristic KR sequence motifs (LXD). In B-type KR domains the *si*-face of the β-carbonyl is reduced, yielding a β-*R* alcohol.^16–18^ However, most similar available KR template structures that could be used for the development of a threaded model are A-type KRs (*e.g.* AmpKR2) and therefore inappropriate.^12,19^ Since the vFAS KR appears to show no substrate chain-length selectivity we also rejected this as a possible template for model construction. We therefore developed an unbiased model of the TENS KR domain using AlphaFold2.^20^

**Fig. 1:**
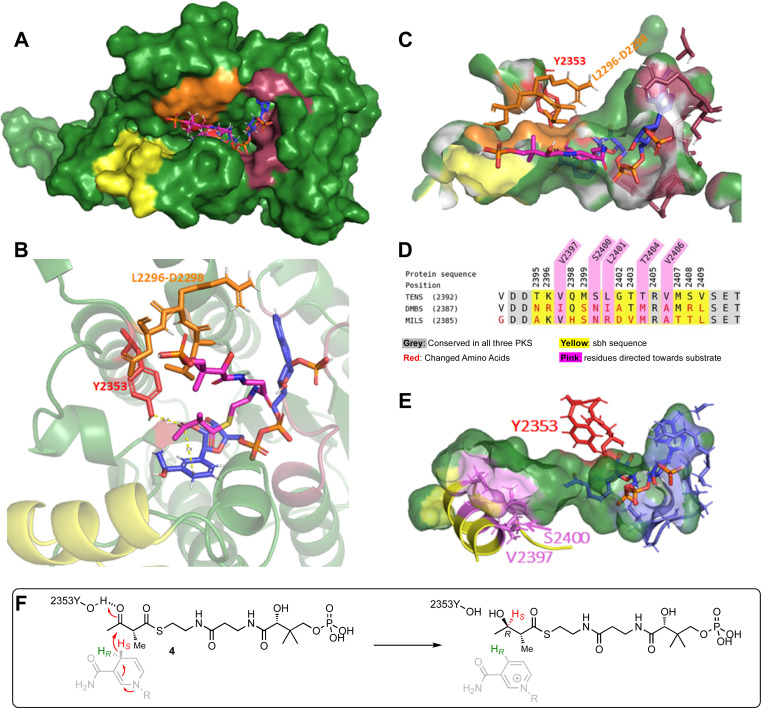
TENS AlphaFold2 structure prediction (red = Y2353, yellow = sbh, blue = NADPH, pink = 2′-methylacetoacetyl pantetheine, orange = LRD motif (L2296–D2298), dark red = NADPH binding residues (2214–2220, S2239, R2240, N2241, D2265, K2316, S2339, N2357, V2382)): (A) TENS KR domain; (B) KR domain active site interaction with docked substrate and cofactor; (C) active site pocket surface view; (D) sequence alignment of TENS, DMBS, and MILS in the substrate binding helix region; (E) visualisation of amino acids of the substrate binding helix in direction towards the substrate (violet); (F) substrate model 4 placement relative to NADPH that would result in B-type keto-reduction.

The AlphaFold2 structure prediction was carried out with the TENS KR amino acid sequence using ChimeraX software on default settings.^20–22^ The structural prediction of the TENS KR (Fig. 1A) was evaluated using the recently published cryo-EM structure of the LovB KR domain (identifier 7cpx), which is the only protein structure of a fungal iterative hr-PKS available.^4^ The AlphaFold KR model and the experimentally obtained LovB KR structure were aligned using PyMOL.^23^ The observed RMSD of 0.90 Å between the TENS model and the LovB cryo-EM structure indicates a likely good model (ESI Fig. S2, Table S5†). The coordinates of the NADPH cofactor were transferred from AmpKR2 (identifier 5xwv) to the TENS KR model.^24^ The *holo*-KR structure was then optimised using the YASARA forcefield.^25,26^ The binding interactions for NADPH (Fig. 1C, dark red) conform to previously observed KR structures, such as LovB and the modular KR AmpKR2.^4,19^ NADPH is bound by the typical Rossmann fold cofactor binding motif (GAAGGLG, positions 2214–2220). D2265 interacts with the adenine of NADPH. Hydrogen bonds established by S2239 and N2241 bind the adenine ribose moiety in place, while R2240 binds to the phosphate *via* a salt bridge.^4,19,27^ The conserved residues S2339, N2357 and K2316 interact with the nicotinamide ribose moiety. Additionally, V2382 facilitates hydrophobic interactions with the nicotinamide aromatic ring, consistent with previously reported KR structures.^4,19^

Manual substrate docking was then performed (Fig. 1A–C). The substrate analogue α-methylacetoacetyl pantetheine 4 was built in Chem3D (PerkinElmer) and inserted into the KR domain structure in PyMOL, while entering the active site from the correct direction according to B-type KR models for the attack of the *si* face of the 3-keto group. While the positions of the protein residues and the cofactor were unchanged, the substrate was fitted into the active site pocket, and manually adjusted. The fitted substrate and the protein structure were refined with YASARA.^25,26^ Reasonable distances were achieved *i.e.* the β-carbon is located 3.0 Å from the 4′-*pro-S* NADPH hydride and the carbonyl oxygen of the β-position is located 1.8 Å from the catalytic tyrosine (Y2353, Fig. 1B, red, *i.e*. *O–O* distance 2.8 Å consistent with the required hydrogen bond at this position). The orientation of the substrate would lead to the formation of a β-*R*-hydroxy stereocentre, in agreement with the known stereochemical outcome of B-type KR domains (Fig. 1F). The LRD-motif, which is typically found in B-type KR domains (Fig. 1, orange), is placed at the expected position adjacent to the active site and the modelled substrate 4. The position of the docked substrate, while consistent with stereochemical and mechanistic knowledge, should be regarded as a representation of a possible pose, which can be used for the development of hypotheses for rational mutation experiments, but conclusions on the final substrate position require experimental confirmation.

Analysis of this model structure showed that the previously identified 69- and 12-amino acid exchange-sequences that appear to govern chain-length selectivity are located adjacent to the active site and include an α-helix. This helix (T2395 to V2409, Fig. 1D, yellow) corresponds to the *lid-helix* in the known structures of modular PKS KR domains that is thought to clamp over bound polyketide intermediates during catalysis.^16,17^ Here we refer to this sequence as the substrate-binding helix (sbh).

Experimental data of B-type KRs shows a more open active site than A-type KR domains, consistent with the AlphaFold model developed above.^28^ The substrate binding helix is part of the active site architecture, which makes it a reasonable target for mutations.^12^ The model of the docked substrate in the TENS KR active site developed above suggests that contact of the substrate binding helix to the substrate could be possible during catalysis, especially for longer polyketide chains which could reach deeper into the active site binding pocket. Such contacts might be significant components of the intrinsic substrate selectivity of the KR domain.

To investigate the potential relevance of the substrate binding helix to substrate selectivity, AlphaFold protein models were also constructed for the KR domains of the related systems DMBS and MILS (ESI Fig. S3†). However, the comparison of TENS, DMBS, and MILS KR domain structures yielded limited insights into differences in programming of the related systems, as no significant differences in the helix positioning were apparent, despite a higher occurrence of exchanged amino acids in comparison to other regions (ESI†). However, prediction of the KR domain structure of vFAS (2vz8, ESI Fig. S3†) revealed that the unprogrammed vFAS possesses an unstructured loop at this position, compared to highly structured helices predicted for the corresponding positions of TENS, DMBS, and MILS.

### 15-Residue swap spanning the substrate binding helix

The Alphafold model demonstrates the close proximity of the substrate binding helix to the substrate during reaction (Fig. 1, yellow), but the open nature of the active site model does not give specific details for contacts between substrate and amino acids. We conducted fragment swaps of residues T2395 to V2409 to probe the relevance of the helix to PKS programming. This was achieved by synthesising two different replacement sequences, which were individually inserted into *tenS* (contained in the entry vector pEYA-*tenS*) by removing an approx. 6400 bp fragment that spans the helix region, and then rebuilding this sequence using native and mutant sequences by rapid recombination in yeast (ESI, Fig. S1†). The incorporation of each mutation was confirmed by sequencing. Each modified *tenS* sequence was then inserted into the expression vector pTY–*argB–tenS–tenC* by Gateway *in vitro* recombination.^29^ The expression vector already contains *tenC* so that transformation into the *Aspergillus oryzae* NSAR1 host achieves the required co-expression of *tenS* and *tenC*.

**Fig. 2:**
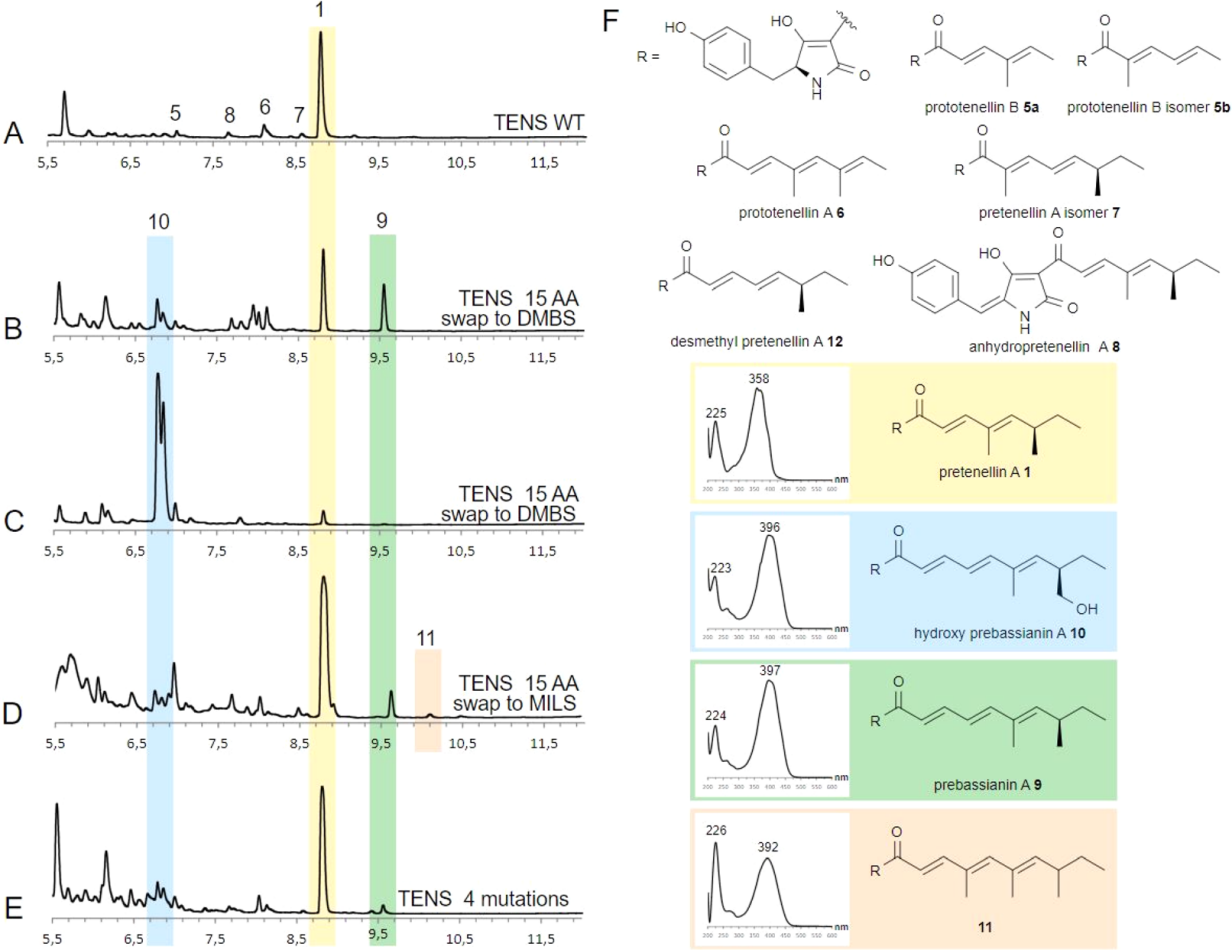
DAD TIC (200–600 nm) chromatogram analysis of TENS variants: (A) TENS WT; (B) TENS sbh = DMBS example 1; (C) TENS sbh = DMBS example 2; (D) TENS sbh = MILS; (E) TENS S2400N, L2401R, T2404M, V2406A; (F) products of TENS WT and TENS variants. See ESI† for expansions of spectra.

Several *A. oryzae* transformants for each system were selected, confirmed by PCR, and then grown in liquid medium under pretenellin A 1 producing conditions. The cultures were extracted with organic solvent, and the organic extracts examined by LCMS. Each of the mutant systems was treated in the same way. In the case of the unmodified system, pretenellin A 1 was the main component, together with known tetra- or pentaketide biproducts 5–8 (Fig. 2A and F), as expected.

Seven transformants containing *tenS(Δsbh:DmbS-sbh)* were obtained of which all produced pretenellin A 1 corresponding to the wild type TENS. Additionally, two related new peaks were observed at 6.8 min and 9.6 min in each transformant with differing ratios (Fig. 2B and C). The MS fragmentation of compound 9 in the ES^−^ and ES^+^ mode suggests a mass of 381 Da at 9.6 min.

Additionally, UV spectra with maxima at 224 nm and 397 nm identified the compound as the doubly methylated hexaketide prebassianin 9 by comparing the data to previous obtained spectra from our group (Fig. 2F).^12^ The compound eluting at 6.8 min was identified similarly by the characteristic data as the known hydroxylated shunt compound hydroxy prebassianin 10, most likely derived from oxygenation of 9 by *A. oryzae* NSAR1 (see ESI† for detailed LCMS analysis).

All producing transformants were screened for previously characterized related polyketides with varying chain length and methylation patterns using the known molecular masses and UV maxima from previous studies (ESI, Table S6†).^12^ Traces of different pentaketides were found, including prototenellin A 6 and the pretenellin A isomer 7, but no evidence for heptaketides was observed.

The ratio between pentaketide 1 and hexaketides (9 and 10 combined) was determined by integration of ELSD chromatograms. Considering all producing transformants, peak integration shows that hexaketides are at least 50% of the total, and up to 97% hydroxy prebassianin 10 in the highest case (Fig. 2C). On average approx. 12% pentaketide pretenellin A 1 is produced in comparison to approx. 88% hexaketides 9 and 10.

The same sequence (T2395 to V2409, 15 residues) was swapped to the corresponding MILS sequence (*tenS(Δsbh:MilS-sbh)*). According to previous results, a swap of 12 residues led to the production of heptaketides when swapping Q2398–V2409.^12^ Therefore, a similar result was expected.

The four mutants containing the substrate binding helix of MILS produced mostly the pentaketide pretenellin A 1 and the hexaketide prebassianin 9 (Fig. 2D), corresponding to the complete substrate helix swap with DMBS. The hydroxylated hexaketide 10 was observed in traces. Additionally, a minor peak was detected at 10.1 min (Fig. 2D). The combination of the mass of 395 Da and an UV adsorption maximum of 392 nm, indicates triple-methylated hexaketide trienone 11.

The presence of 9, 10 and 11 are indications that TENS was reprogrammed to produce hexaketides, but no heptaketides were detectable. In a previously reported swap of positions Q2398–V2409 to MILS amino acids, heptaketides were observed in low titres, but as the dominant overall-product.^12^ Only one additional residue was exchanged in case of the experiment of this work. Position K2396 and V2397 are conserved in both KR domains (Fig. 1D). Thus, the only non-conservative mutation included in position 2305 to 2307 is the mutation T2395A. Position T2395 is therefore an interesting focus for future work. The swap to the MILS sequence is less effective in terms of changes in the chain length than the swap to the DMBS sequence, based on the achieved titres of unnatural products. However, 11 indicates an effect on the methylation programming by the T2395 to V2409 swap.

### 4-Residue swap within the substrate binding helix region

The AlphaFold model suggests that the side chains of residues V2397, S2400, L2401, T2404 and V2406 are directed towards the active site of the KR active site pocket (Fig. 1E, violet), which could indicate possible role for substrate contact and selectivity. Other amino acids of the sequence point mainly to the bottom side of the pocket or to the opposite side (Fig. 1D and E, yellow) and therefore are not in focus for amino acid swaps, although these positions of the helix are also changed between the different hr-PKS (Fig. 1D and E, yellow).

Positions V2397, S2400, L2401, T2404 and V2406 were further evaluated based on sequence alignment to select the appropriate mutations (Fig. 1D and E). Residue V2397 is in putative close contact to the substrate. However, position V2397 was not included in the rational engineering approach, as in two PKS (TENS and MILS) the amino acid is conserved and for DMBS a conservative replacement by isoleucine is present at this position (Fig. 1D). Residue L2401 is in both DMBS and MILS swapped to different amino acids. Leucine (TENS) and isoleucine (DMBS) are structurally similar, since both are aliphatic C_4_-residues. Thus, the non-conservative arginine residue mutation was chosen for swapping experiments (Fig. 1D). Hence, the selection of the rational engineering of the PKS includes the four mutations S2400N, L2401R, T2404M and V2406A. These changes were made in a short synthetic DNA fragment that was assembled into the complete *tenS* sequence as previously described.

Five producing transformants containing the 4-residue change were analysed as described previously. All five of the transformants showed pretenellin A 1 as the main product (Fig. 2E). Additionally, a minor amount of prebassianin A 9 and hydroxy prebassianin A 10 were identified (Fig. 2E). 9 and 10 were detected in three producing transformants in extracted ion chromatograms. No additional new compounds were found.

### Alanine scan through the substrate binding helix

Finally, an alanine scan was performed to find single amino acids of the substrate binding helix, which play a crucial role in the KR domain programming. Therefore, 15 amino acids of the KR domain from TENS were exchanged to alanine (between D2394 to S2410) in individual experiments by previously described methods.

The results of the swaps of position S2400A, L2401A, T2404A and V2406A are especially of interest, as they were hypothesized to be important, based on the KR model in the prior analysis (Fig. 1E).

**Fig. 3:**
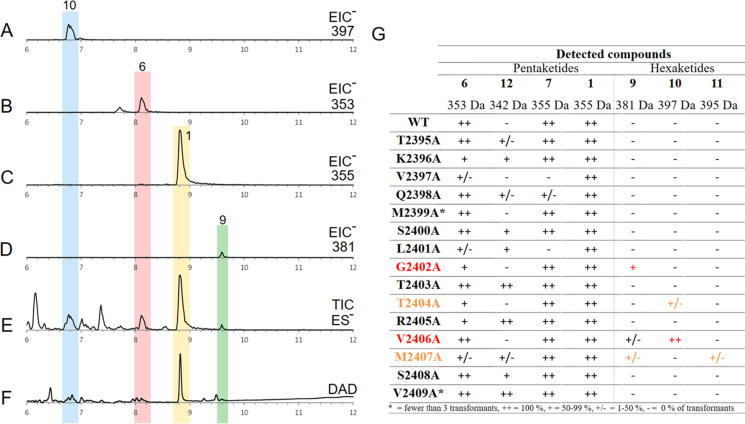
Analysis of *in vivo* alanine scan: (A) EIC ES^−^ for mass 397 of transformant V2406A; (B) EIC ES^−^ for mass 353 of transformant V2406A; (C) EIC ES^−^ for mass 355 of transformant V2406A; (D) EIC ES^−^ for mass 381 of transformant V2406A; (E) TIC ES^−^ of transformant V2406A; (F) DAD chromatogram (200–600 nm) of transformant V2406A; (G) overall analysis of detected compounds from alanine scan experiments.

All transformants from all 15 alanine scan mutants produced pretenellin A 1 as the main product. However, traces of additional related compounds (6, 7, 12) were detected by searching for extracted ion chromatogram signals for known compounds (Fig. 3G). For example, a run with the TENS variant V2406A (Fig. 3A–F) included traces of the hexaketide structures prebassianin 9 and hydroxyl prebassianin 10 (Fig. 3A and D) in addition to pretenellin A 1 as the main product and the pentaketide 6 (Fig. 3B, C and E, F).

Hexaketide compounds were found in extracts of transformants including the following mutations (Fig. 3F): G2402A (in 5 from 9 extracts), T2404A (in 1 of 7 extracts), V2406A (in 3 from 3 extracts), M2407A (in 1 from 5 extracts). However, these results are associated with very low concentration of the hexaketides in each analysis in comparison to pretenellin A 1 production. Not only has the level of production significance for the reliability of the results, but also the consideration of all producing transformants of one alanine scan position. In the case of the mutations at positions T2404A and M2407A, only one transformant was observed to produce traces of hexaketides, which indicates less reliable results. At positions V2406A and G2402A, the results were more consistent for the production of hexaketides. In the case of V2406A, hexaketides were detected in 100% of the cases. For position G2402A, 55% hexaketide producing transformants were observed. Thus, these positions are suggested to be particularly important for the programming of the PKS.

## Discussion and conclusion

An AlphaFold2 protein model of the TENS KR domain was generated and used for simulated docking of a substrate pantetheine 4. Although the docked substrate appeared to take up a reasonable pose in the active site, interacting with the cofactor and known catalytic residues that would result in the correct stereoselectivity, the model likely represents an early binding pose prior to catalysis. It has been suggested that the substrate binding helix (lid motif) of KR domains clamps down on the substrate during reaction,^17^ but this did not seem to be apparent in the rather open AlphaFold2 model. For example the KR1 structure from the bacterial tylosin modular PKS^17^ has the substrate-binding helix (lid helix) located much closer to the active site residues: *e.g*. 21.3 Å between the amide nitrogens of T2395 and Y2353 in the TENS KR model *vs.* 13.0 Å for the corresponding residues of tylosin KR1 (ESI Fig. S4†). While we can reasonably speculate that the sbh is likely to contact the substrate *during* reaction, the model generated here cannot give sufficient detail of the specific interactions, or precisely define limitations in a potential binding pocket.

However, initial experiments in which 15 residues of the putative TENS sbh (T2395 to V2409 inclusive) were exchanged with corresponding residues from the DMBS (hexaketide) and MILS (heptaketide) systems did clearly result in large changes in the chain-length selectivity of TENS, in agreement with previous observations.^12^ In the normal situation, pentaketides are the overwhelmingly dominant product of TENS, but in these swaps up to 97% of the observed products were hexaketides. Interestingly, these experiments revealed a transformant-by-transformant variation in the mixtures of products produced by ostensibly similar systems. We interpret this to perhaps indicate that different transformants may display different ratios of *tenS* and *tenC* expression. Our previous work has shown that the level of *tenC* expression can affect the programming of the TENS/TENC system.^9^ This complicates the interpretation of the *in vivo* results, especially where subtle changes are observed, and so only broad conclusions can be drawn from the following experiments.

Inspection of the AlphaFold2 model suggested that residues S2400, L2401, T2404 and V2406 might contact the substrate during reaction. To test this idea a quadruple mutant was generated (S2400N, L2401R, T2404M and V2406A), swapping each position to the cognate residue from the MILS heptaketide synthase. The results showed this mutant is able to produce hexaketides whereas the wild-type system cannot. However the production is significantly less than hexaketide production by the full sbh swaps, and only makes up *ca.* 1.6% of their total.

Finally, an alanine scan experiment was attempted in which each and every position between T2395 and V2409 inclusive was exchanged for alanine. The results of these experiments showed that no single position exercises a high level of control over the reprogramming. Where hexaketides were observed it was always in very low titres. The only consistently reliable producer of hexaketides was the V2406A mutant, but the level of production was consistently less than the production of hexaketides by the 4-way mutation or the full sbh swaps. This observation is reminiscent of engineering efforts in other Type I PKS systems. For example, Williams, Sherman and colleagues showed that a *motif-swapping* strategy was much more effective for engineering the selectivity of AT domains from the erythromycin modular PKS as compared to the use of single residue mutations.^30^

Previous experimental results from our laboratories and others have begun to reveal the cryptic programming of iterative polyketide and fatty acid synthases. The consensus view is that at certain points during chain construction, kinetic factors guide the programming choices. We have divided these factors into intrinsic causes (*e.g*. active-site changes that might affect recognition and the rate of catalysis); and extrinsic features (*e.g.* possible dynamic effects). In this study we attempted to try to link these programming decisions with specific structural features of the TENS PKS. In particular, we hypothesised that *intrinsic* structural features of the KR could contribute to the rate at which the KR competes with off-loading. When the KR is fast and ‘wins’ such a competition then the growing polyketide is committed to further processing and eventual chain extension. But if the off-loading out-competes the KR, then the β-ketoacyl intermediate is transferred to the NRPS for cyclisation and release. Our experiment shows that affecting the rate of the KR is no simple matter, as single and 4-way mutations made either no significant, or marginal, differences. However, exchange of the full substrate-binding helix can make a major change. For example, change to the DMBS sequence involves exchange of 11 out of 15 residues and this can, in turn, lead to significant reprogramming of the PKS products to hexaketides.

These observations have important implications for future reprogramming and engineering efforts focussed on iterative systems. First, static simple models of the catalytic domains are poorly able to model intrinsic substrate binding, and certainly cannot offer insight into extrinsic dynamic processes. For example, here we cannot distinguish whether substrate-recognition and binding (intrinsic), or helix dynamic (extrinsic) properties are at the root cause of reprogramming. Second, it is clear that reprogramming results from an accumulation of small changes. Here we examined changes in a helix that is likely to play a role close to the substrate during catalysis, but our results find no distinct ‘programming switch’ located in a single residue. Finally our results suggest that future attempts to engineer these types of iterative synthases may be more successful if they focus on larger domain swaps (*e.g.* 3° motifs) rather than attempts to engineer individual 2° structural motifs or individual residues. Our observations also raise interesting questions about the evolution of programming in such iterative systems when it appears that for mutations to have significant effects on product structure, they must be widespread and cooperative.

## Author contributions

The project was devised by RJC and KS. KS performed all experimental work and results were analysed by RJC and KS. KS drafted the manuscript which was polished by both authors.

## Conflicts of interest

RJC is joint Editor-in-chief of *RSC Advances*.

## Supplementary Material

RA-014-D3RA08463A-s001
