# Supplementary material for: Investigation of chain-length selection by the tenellin iterative highly-reducing polyketide synthase
Source: RSC Adv. 2024 Mar 15;14(13):8963–70. doi: 10.1039/d3ra08463a (PMC10941261; doi:10.1039/d3ra08463a)
Supplement: RA-014-D3RA08463A-s001 [file RA-014-D3RA08463A-s001.pdf]

Electronic Supplementary Material (ESI) for RSC Advances.
This journal is © The Royal Society of Chemistry 2024

Investigation of Chain-Length Selection by the Tenellin Iterative Highly-Reducing Polyketide Synthase

Katharina Schmidt and Russell J. Cox

BMWZ and Institute for Organic Chemistry

Leibniz Universität Hannover

Schneiderberg 38

30167 Hannover, Germany

Electronic Supplementary Information

Content

1 Methods 3
1.1 Cloning Procedure, Vectors and Oligonucleotides 3
1.2 Strains and Cultivation 5
1.2.1 Growth and Maintenance 7
1.2.2 Transformations 7
1.3 Analytical LCMS 7
2 Results 8
2.1 Protein Structures 8
2.1.1 Comparison of TENS model and LOVB Structure 8
2.1.2 Protein Models of TENS, DMBS, MILS, and vFAS 9
2.2 Spectroscopic Data for Identified Compounds 11
2.2.1 Detailed Analysis of Compound 10 12
2.3 Overview of all Generated Transformants 16
3 Multiple Alignment 18
4 References 19

1 Methods

1.1 Cloning Procedure, Vectors and Oligonucleotides

The strategy for vector construction (Figure S1) was based on yeast homologous recombination. The enzymes (EcoRI, FseI) used for vector digestion within the *tenS* gene were purchased from New England Biolabs (Beverly, MA, USA) and used according to the manufacturer's instructions with appropriate supplied buffers.

The proofreading Q5® 2x Master Mix (New England Biolabs) was used to obtain DNA fragments needed for further cloning procedure (F1-F3) with the pEYA-*tenS* as a template. The information provided by the manufacturer served as a template. Mutations/swap sequences were introduced by synthetic fragments (Twist Bioscience) to rebuild pEYA-*tenS**.

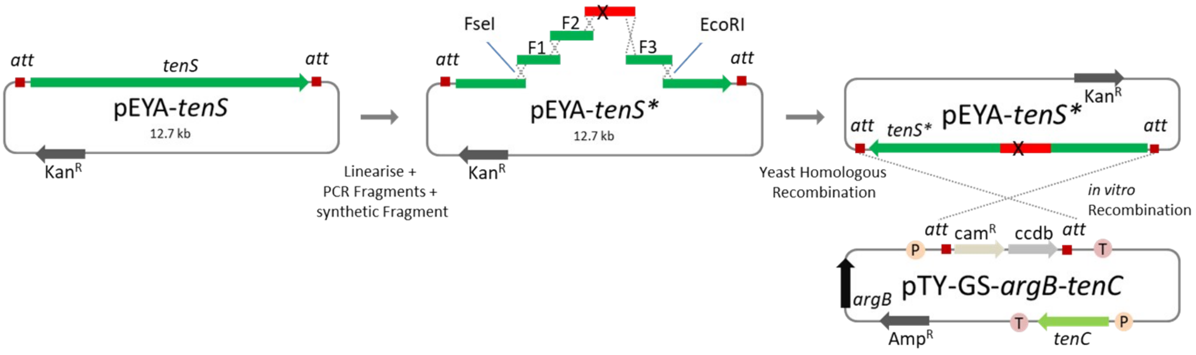

Figure S1 Cloning strategy.

The transformation of *S. cerevisiae* was done using the LiOAc/SS carrier DNA/PEG protocol developed by Gietz and Woods.[1,2] Therefore, fresh yeast cells were incubated with the transformation-mix (50 µl 2 mg/ml ssDNA, 240 µl 50 % PEG 3350, 36 µl 1 M LiAc, up to 5 µg DNA (cut plasmid, fragments, equimolar)) for 42 °C for 50 min. Cells were pelleted at 11000 x g for 30 s and re-suspended with 500 µl water. 250 µl of the cell mixture was spread on selective SM-URA plates and incubated for 3-5 days at 30 °C. The vector DNA was then purified from yeast, transformed into *E. coli* Top 10. DNA samples were sequenced by *Eurofins Genomics* (Mix2Seq OVERNIGHT, Ebersberg).

Gateway LR Clonase II Enzyme mix kit (Invitrogen)[3] was used to transfer genes from the entry vector pEYA-*tenS** to the destination vector pTY-GS-*argB*-*tenC* (Figure S1). The manufacturer's instructions were followed. For *E.coli* Top10 transformation, the vector mixture (10 µl) was added to 50 µl competent cells.

Information about the used vectors in this work are summarised in Table S1.

Table S1 Summary of used vectors.

Name	Description	Selection
pEYA-*tenS*	pEYA shuttle vector including *tenS* gene	Kan^R
pTY-*argB-tenC*	fungal expression vector with trans-ER *tenC* gene	*argB*, arb^R, *URA3*
pTY-*argB-tenS-tenC*	pTY-*argB-tenC* including *tenS* gene	*argB,* arb^R, *URA3*
pEYA-*tenS** sbh:DmbS	T2395 to V2409 swap to DmbS sequence	Kan^R
pEYA-*tenS** sbh:MilS	T2395 to V2409 swap to MilS sequence	Kan^R
pEYA-*tenS** 2400N, L2401R, 2404M, V2406A	Mutations 2400N, L2401R, 2404M, V2406A	Kan^R
pTY-*argB-tenS*-tenC* sbh:DmbS	fungal expression vector with trans-ER *tenC* gene, T2395 to V2409 swap to DmbS sequence	*argB,* arb^R, *URA3*
pTY-*argB-tenS*-tenC* sbh:MilS	fungal expression vector with trans-ER *tenC* gene, T2395 to V2409 swap to MilS sequence	*argB,* arb^R, *URA3*
pTY-*argB-tenS*-tenC* 2400N, L2401R, 2404M, V2406A	fungal expression vector with trans-ER *tenC* gene, mutations 2400N, L2401R, 2404M, V2406A	*argB,* Carb^R, U*RA3*
pEYA-*tenS** AlaN	pEYA-tenS with alanine muatations at position N (N between D2394 to S2410)	Kan^R
pTY-*argB-tenS**AlaN-*tenC*	pTY-*argB-tenC* including *tenS* gene alanine muatations at position N (N between D2394 to S2410)	*argB,* Carb^R, *URA3*

Information about the used oligonucleotides in this work are summarised in Table S2. They were synthesized by Sigma Aldrich and supplied lyophilised. They were dissolved according to the manufacturer's instructions.

Table S2 Summary of used oligonucleotides.

Description	Name	Sequence
Fragment 1	A1	CGCATTCTCCACGGCTATTGGAAAC
	A2	CCTGCTGATCTTCCTGAACGTCG
Fragment 2	A3	GATGCCCAGCTCCAAAAGGCC
	A4	CTGTATTATTCAGAATGGCAGCGCTCGAGCTTAGCAAGACAAAAAAGTC
Fragment 3	A5	CGGCTCCCACAACATCATAATGG
	A6	GCTTTGGACGATGCGGCGCGG
Fragment 2/3 for alanine scan	A4_alascan	GTCGTCAACCAAGCGGGCAAC
	A5_alascan	GTCGTCAACCAAGCGGGCAAC
synthetic fragments including mutations	sbh = DmbS	GCTCGAGCGCTGCCATTCTGAATAATACAGGCCAGTCAAACTACCACTGCGCAAATCTCTA<br>CATGGACAGCCTGGTCACCAATCGGCGCTCGAGAGGACTCGCAGCTTCCATTATCCATATC<br>GGTCATGTCTGCGACACGGGATACGTTGCCCGCTTGGTTGACGACACCAAGGTGCAGATG<br>AACCTAGGTACCATGCGAGCCATGAGTGTCTCTGAGACGGATGTGCATCATGCCTTTGCT<br>GAGGCGGTCCGCGGGGGGCAGCCAGACAGCCGGAGCGGCTCCCACAACATCATAATGG<br>GTATTGA

synthetic fragments including mutations	sbh = MilS	GCTCGAGCGCTGCCATTGCGAATAATACAGGCCAGTCAAACTACCACTGCGCAAATCTCTACATGGACAGCCTGGTCACCAATCGGCGCTCGAGAGGACTCGCAGCTTCCATTATCCATATCGGTCATGTCTGCGACACGGGATACGTTGCCCGCTTGGTTGACGACACCAAGGTGCAGATGAACCGAGGTACCATGCGAGCCATGAGTGTCTCTGAGACGGATGTGCATCATGCCTTTGCTGAGGCGGTCCGCGGGGGGCAGCCAGACAGCCGGAGCGGCTCCCACAACATCATAATGGGTATTGA
synthetic fragments including alanine-swaps for alanine scan	Ala1	ACGGGATACGTTGCCCGCTTGGTTGACGACGCCAAGGTGCAGATGAGCCTAGGTACCACGCGAGTCATGAGTGTCTCTGAGACGGATGTGCATCATGCCTTTGCT
	Ala2	ACGGGATACGTTGCCCGCTTGGTTGACGACACCGCGGTGCAGATGAGCCTAGGTACCACGCGAGTCATGAGTGTCTCTGAGACGGATGTGCATCATGCCTTTGCT
	Ala3	ACGGGATACGTTGCCCGCTTGGTTGACGACACCAAGGCGCAGATGAGCCTAGGTACCACGCGAGTCATGAGTGTCTCTGAGACGGATGTGCATCATGCCTTTGCT
	Ala4	ACGGGATACGTTGCCCGCTTGGTTGACGACACCAAGGTGGCGATGAGCCTAGGTACCACGCGAGTCATGAGTGTCTCTGAGACGGATGTGCATCATGCCTTTGCT
	Ala5	ACGGGATACGTTGCCCGCTTGGTTGACGACACCAAGGTGCAGGCGAGCCTAGGTACCACGCGAGTCATGAGTGTCTCTGAGACGGATGTGCATCATGCCTTTGCT
	Ala6	ACGGGATACGTTGCCCGCTTGGTTGACGACACCAAGGTGCAGATGGCCCTAGGTACCACGCGAGTCATGAGTGTCTCTGAGACGGATGTGCATCATGCCTTTGCT
	Ala7	ACGGGATACGTTGCCCGCTTGGTTGACGACACCAAGGTGCAGATGAGCGCAGGTACCACGCGAGTCATGAGTGTCTCTGAGACGGATGTGCATCATGCCTTTGCT
	Ala8	ACGGGATACGTTGCCCGCTTGGTTGACGACACCAAGGTGCAGATGAGCCTAGCTACCACGCGAGTCATGAGTGTCTCTGAGACGGATGTGCATCATGCCTTTGCT
	Ala9	ACGGGATACGTTGCCCGCTTGGTTGACGACACCAAGGTGCAGATGAGCCTAGGTGCCACGCGAGTCATGAGTGTCTCTGAGACGGATGTGCATCATGCCTTTGCT
	Ala10	ACGGGATACGTTGCCCGCTTGGTTGACGACACCAAGGTGCAGATGAGCCTAGGTACCGCGCGAGTCATGAGTGTCTCTGAGACGGATGTGCATCATGCCTTTGCT
	Ala11	ACGGGATACGTTGCCCGCTTGGTTGACGACACCAAGGTGCAGATGAGCCTAGGTACCACGGCAGTCATGAGTGTCTCTGAGACGGATGTGCATCATGCCTTTGCT
	Ala12	ACGGGATACGTTGCCCGCTTGGTTGACGACACCAAGGTGCAGATGAGCCTAGGTACCACGCGAGCCATGAGTGTCTCTGAGACGGATGTGCATCATGCCTTTGCT
	Ala13	ACGGGATACGTTGCCCGCTTGGTTGACGACACCAAGGTGCAGATGAGCCTAGGTACCACGCGAGTCGCGAGTGTCTCTGAGACGGATGTGCATCATGCCTTTGCT
	Ala14	ACGGGATACGTTGCCCGCTTGGTTGACGACACCAAGGTGCAGATGAGCCTAGGTACCACGCGAGTCATGGCTGTCTCTGAGACGGATGTGCATCATGCCTTTGCT
	Ala15	ACGGGATACGTTGCCCGCTTGGTTGACGACACCAAGGTGCAGATGAGCCTAGGTACCACGCGAGTCATGAGTGCCTCTGAGACGGATGTGCATCATGCCTTTGCT
primer for control and sequencing	KR_amp_fw	ATGGTCTTGCGTGACAAGCTT
	KR_amp_rev	CATCTGATTCTCCAGGGTGCTAAA
	SQ_KR	GCAAGGTACGGAGCATCTGGACTCG

1.2 Strains and Cultivation

Information about the used strains in this work are summarised in Table S3.

Table S3 Summary of used strains.

Organism	Strain	Genotype	Reference
E. coli	OneShot® Top10	*F^- mcrA Δ(mrr-hsdRMS-mcrBC) Φ80lacZΔM15 Δ lacX74 recA1 araD139 Δ(araleu)7697 galU galK rpsL (StrR) endA1 nupG*	Thermo Fisher Scientific
	OneShot® ccdB survival 2T1^R	*F^-mcrA Δ(mrr-hsdRMS-mcrBC) Φ80lacZΔM15 ΔlacX74 recA1 araΔ139 Δ(ara-leu)7697 galU galK rpsL (Str^R) endA1 nupGfhuA::IS2*	Thermo Fisher Scientific
S. cerevisiae	CEN.PK2	*MATa/a ura3-52/ura3-52trp1-289/trp1-289 leu2-3_112/leu2-3_112 his3D1/his3 D1MAL2-8C/MAL2-8C SUC2/SUC2*	Euroscarf
A. oryzae	NSAR1	*ΔargB sC$^-$ ΔadeA niaD$^-$*	Lazarus group, Bristol

All media (Table S4), buffers and solutions used in this work were prepared with Millipore water (GenPure Pro UV/UF milipore device, *Thermo Fisher Scientific*) and sterilised by autoclaving 15 min at 121 °C (Autoclave 2100 Classic, *Prestige Medical*) or sterilised by disposable syringe filters (pore size 0.2 – 0.4 µm, *Carl Roth*). The pH was adjusted with 2 M HCl or 2 M NaOH by using a FiveEasy Standard pH Meter Line (*Mettler Toledo*).

Table S4 Media used in this work.

Media	Composition [% (w/v)]
CMP media	3.5 % Czapek Dox broth (Duchefa Biochemie), 2 % D(+)-Maltose monohydrate (Duchefa Biochemie), 1 % Polypeptone (Roth), 3.5 % Czapek Dox broth (Duchefa Biochemie)
CZD/S agar	3.5 % Czapek Dox broth (Duchefa Biochemie), 18.22 % D-Sorbitol (=1 M) (Roth), 0.1 % Ammonium sulfate (Roth), 0.05 % Adenine (Roth), 0.15 % L-Methionine (Roth), 1.5 % Agar (Duchefa Biochemie)
CZD/S softagar	3.5 % Czapek Dox broth (Duchefa Biochemie), 18.22 % D-Sorbitol (=1 M) (Roth), 0.1 % Ammonium sulfate (Roth), 0.05 % Adenine (Roth), 0.15 % L-Methionine (Roth), 0.8 % Agar (Duchefa Biochemie)
DPY agar	2 % Dextrin from potato starch (Sigma Aldrich), 1 % Polypeptone (Roth), 0.5 % Yeast extract (Duchefa Biochemie), 0.5 % Monopotassium phosphate (Roth), 0.05 % Magnesium sulfate hexahydrate (Sigma Aldrich), 2.5 % Agar (Duchefa Biochemie)
GN media	2 % D(+)-Glucose Monohydrate (Roth), 1 % Nutrient broth Nr. 2 from Oxoid (Fisher Scientific)
LB agar	0.5 % Yeast extract (Duchefa Biochemie), 1 % Tryptone (Duchefa Biochemie), 0.5 % Sodium chloride (Roth or VWR), 1.5 % Agar (Duchefa Biochemie)
LB media	0.5 % Yeast extract (Duchefa Biochemie), 1 % Tryptone (Duchefa Biochemie), 0.5 % Sodium chloride (Roth or VWR)
SM-URA agar	0.17 % Yeast nitrogen base (Sigma Aldrich), 0.5 % Ammonium sulfate (Roth), 2 % D(+)-Glucose monohydrate (Roth), 0.077 % Complete supplement mixture minus Uracil (Sigma Aldrich), 2.5 % Agar (Duchefa Biochemie)
SOC media	0.5 % Yeast extract (Duchefa Biochemie), 2 % Tryptone (Duchefa Biochemie), 0.06 % Sodium chloride (Roth or VWR), 0.02 % Potassium chloride (Roth), 25 mM Magnesium chloride hexahydrate (Roth), 1 % D(+)-Glucose monohydrate (Roth)
YPAD agar	1 % Yeast extract (Duchefa Biochemie), 2 % Tryptone (Duchefa Biochemie), 2 % D(+)-Glucose monohydrate (Roth), 0.03 % Adenine (Roth), 1.5 % Agar (Duchefa Biochemie)
YPAD media	1 % Yeast extract (Duchefa Biochemie), 2 % Tryptone (Duchefa Biochemie), 2 % D(+)-Glucose monohydrate (Roth), 0.03 % Adenine (Roth)

Antibiotics (carbenicillin, kanamycin) were prepared in 1000x concentrated stock solution in distilled water in a concentration of 50 µM. Stocks were sterilized through 0.45 µm syringe filter and stored at -20 °C. Antibiotics were diluted for a final concentration of 50 µg/ml by adding them to the final media.

1.2.1 Growth and Maintenance

E. coli cells were grown on LB-agar or in liquid LB-medium with corresponding antibiotics. The cells were cultivated at 37 °C for approx. 12 h. If grown in liquid media, the culture was shaking at 200 rpm.

S. cerevisiae cells were grown on solid YPAD agar at 30 °C for 3 – 5 days. One single colony was used for inoculation for 10 ml liquid YPAD medium. The culture was grown overnight at 30 °C and 200 rpm. After transformation of cells with vectors containing *ura3* selection marker the cultivation took place with selective SM-URA-Agar and at 30°C for 3-5 days.

A. oryzae strain NSAR1 was grown on DPY-agar plates for 3-7 days at 28 °C. *A. oryzae* strain NSAR1 transformants were grown in 100 ml CMP liquid medium in 500 ml baffled shake flasks for 6 days at 28 °C and 110 rpm.

1.2.2 Transformations

Competent *E.coli* strains were thawed on ice after - 80 °C storage. 60 – 100 ng purified plasmid was added to 50 µl *E.coli* cells and placed on ice for 30 min, followed by a heat shock at 42 °C for 30 s and cooling on ice for 2 min. 250 µl SOC-medium was added to the cells and the mixture was incubated at 37°C and 300 rpm for 1 h. The transformed cells were spread out on LB-agar plates containing appropriate antibiotics and incubated at 37 °C overnight.

A. oryzae NSAR1 was grown on DPY-plates at 28 °C for 5-7 days. The mycelium was used to inoculate 50 ml GN-medium in 250 ml shake flask. The culture was incubated at 28 °C and 110 rpm for approx. 18 h. The biomass was separated from the media by filtration with a miracloth filter. Mycelia was incubated with VinoTaste® Pro (Novozymes) solution (10 mg/ml enzyme) while shaking at room temperature and 2 rpm for 3-5 hours. The protoplasts were obtained by filtration with a miracloth filter and centrifuged at 3000 x g for 5 min. The resulting pellet was re-suspended with solution 1 (100 µl per transformation, 0.8 M sodium chloride, 10 mM calcium chloride, 50 mM Tris-HCl, pH 7.5). Vector DNA was added to 100 µl protoplast suspension and incubated on ice for 2 min. Then 1 ml of solution 2 (60 % (w/v) PEG 3350, 0.8 M sodium chloride, 10 mM calcium chloride, 50 mM Tris-HCl, pH 7.5) was added to the mixture and incubated at room temperature for 20 min. After incubation 5 ml of appropriate selective softagar was added to the mixture and overlaid over prepared plates with corresponding agar. Plates were incubated at 28 °C for 4-6 days. When mycelia was visible, the transformants undergo two further selection rounds to avoid false-positive transformants. The colonies are picked from the agar, placed on fresh selection agar plates, and grown for 3-5 days. For the preparation of liquid cultures the transformants were grown on DPY agar plates for 5 days. The spores were used to inoculate 100 ml CMP liquid medium.

1.3 Analytical LCMS

Analytical LCMS was run to analyse the extracts from fungal cultures. The Waters LCMS system containing a Waters 2767 autosampler, Waters 2545 pump, a Phenomenex Kinetex column (2.6 µm, C18, 100 Å, 4.6 x 100 mm), a Phenomenex Security Guard precolumn (Luna, C5, 300 Å) was used with a flow rate of 1 ml/min. The equipped detectors were a diode array detector (Waters 2998) in the range 210 to 600 nm and an ELSD detector (Waters 2424) together with a mass spectrometer, Waters SQD-2 mass detector (ES^+ and ES^-, 150 and 1000 m/z). For elution, a solvent gradient was run

for 15 min starting at 10 % acetonitrile/ 90 % HPLC grade water (0.05 % formic acid) and ramping to 90 % acetonitrile.

2 Results

2.1 Protein Structures

2.1.1 Comparison of TENS model and LOVB Structure

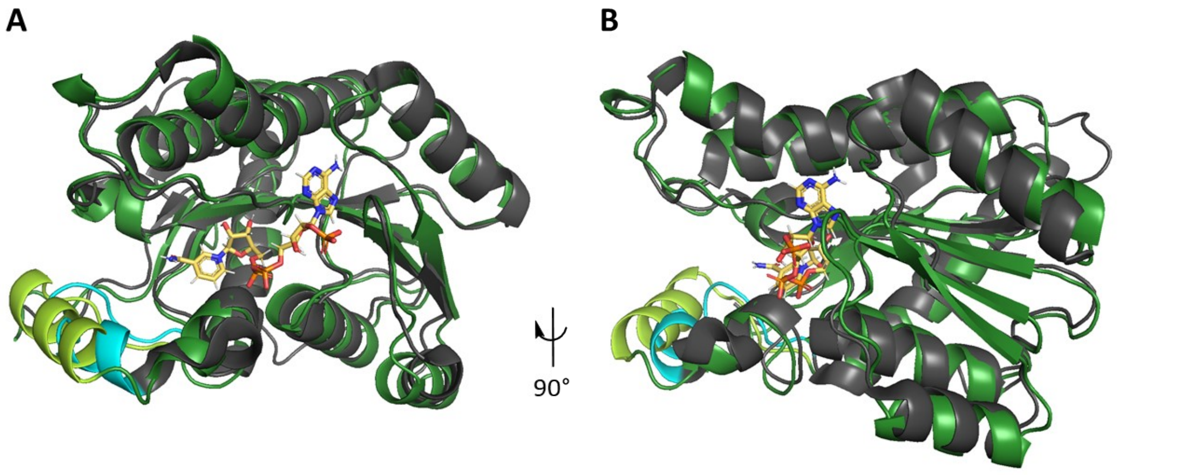

Figure S2 Alignment of TenS KR models to LovB cryo-EM KR structure: **A**, TenS AlphaFold model *vs.* LovB in frontal view; **B**, TenS AlphaFold model *vs.* LovB in side view (grey = LovB, green = AlphaFold model, yellow = cofactor from experimental LovB data).

Table S5 Structural comparison and resulting RMSD of LovB with threaded and AlphaFold model.

compared area	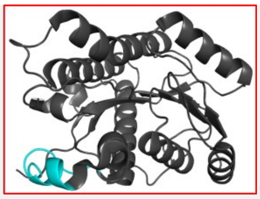 c<br>omplete domain	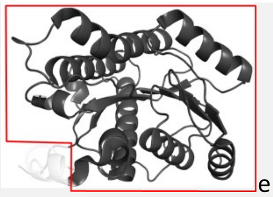 e<br>xcluding sbh	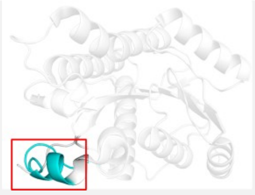 o<br>nly sbh
RMSD of AlphaFold model to LovB [Å]	0.90	0.87	2.81

2.1.2 Protein Models of TENS, DMBS, MILS, and vFAS

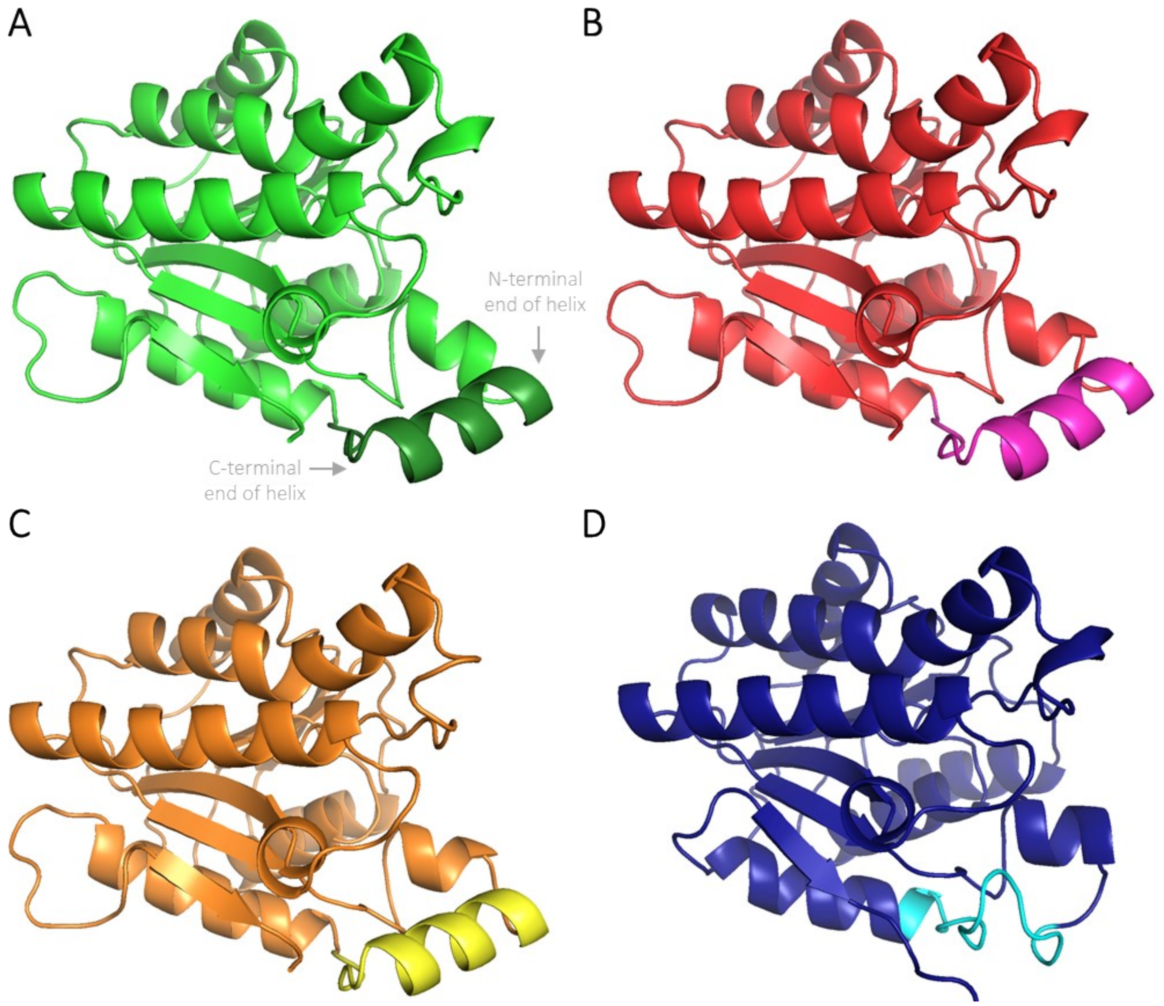

Figure S3 Structural protein models based on AlphaFold: **A**, TenS (sbh dark green); **B**, DmbS (sbh magenta); **C**, MilS (sbh yellow); **D**, mFAS (pig, sbh cyan).

2.1.3 Comparison of sbh in TENS, AmphB and Tylosin KR1 structures

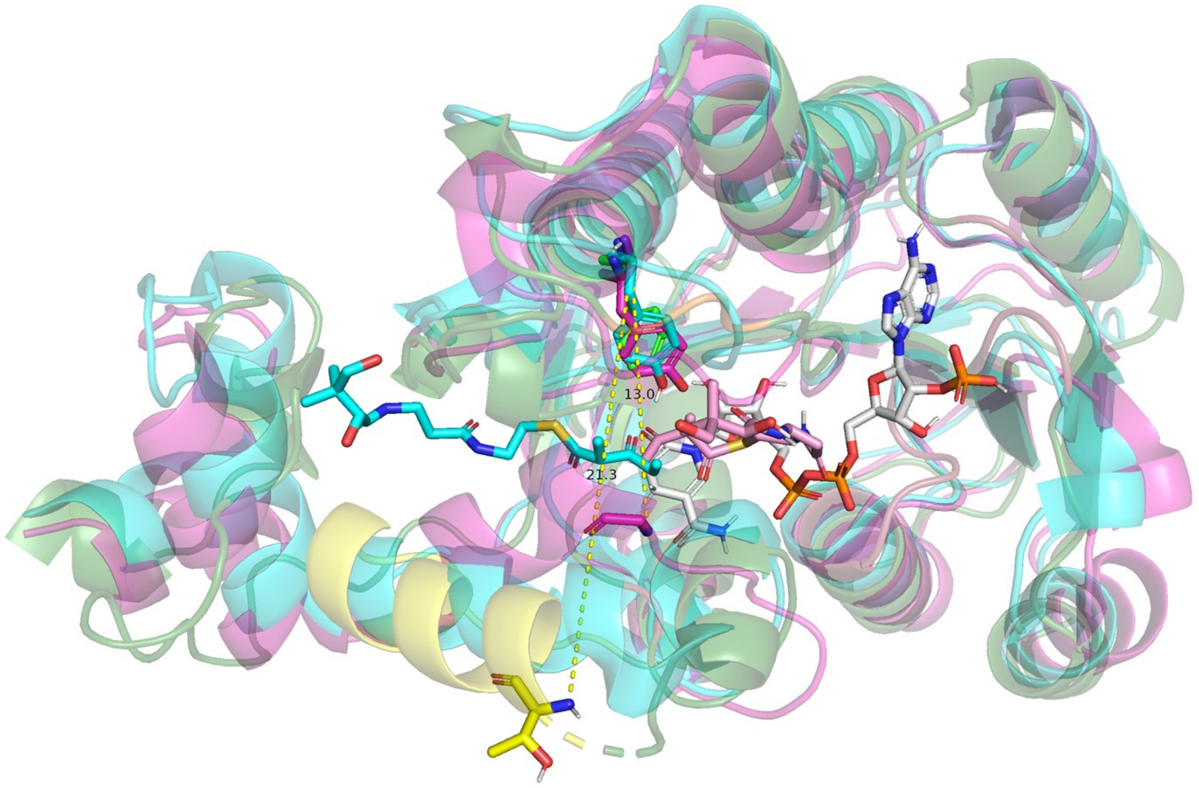

Figure S4. Overlay of TENS KR model (green-yellow), AmphB KR (cyan) and Tylosin KR1 (magenta) showing location of cofactor (grey), Amph B substrate (cyan) and TENS substrate (magenta). The substrate-binding helix (sbh) of the TENS KR is indicated in yellow and overlays well with the 'lid-helix' of the Amph B structure, but the lid-helix of the Tylosin structure approaches the bound substrates more closely. Distances calculated between amide nitrogen of N-terminal residues of the helices and the amide nitrogen of the active site tyrosine.

2.2 Spectroscopic Data for Identified Compounds

Table S6 Overview of UV-, ES⁻-, ES⁺-spectra for detected compounds.

No	Structure	UV spectrum	ES⁻	ES⁺
**1**	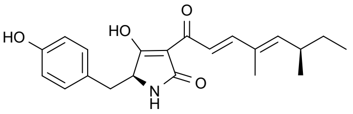 pretenellin A<br>Mass: 355.18	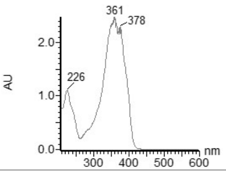	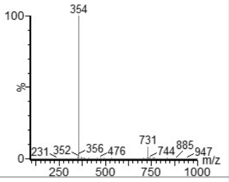	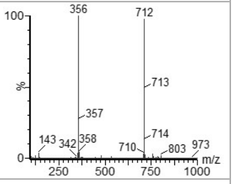
**4/5**	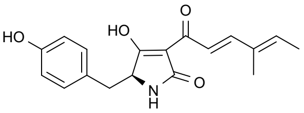 prototenellin B<br>Mass: 313.13<br>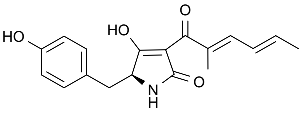 prototenellin B isomer<br>Mass: 313.13	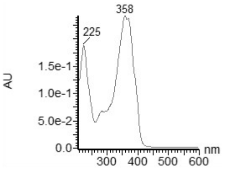	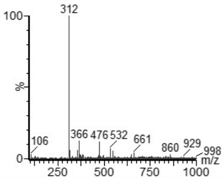	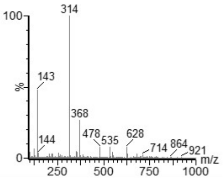
**6**	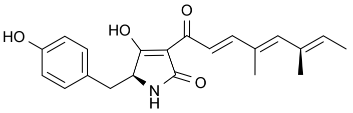 prototenellin A<br>Mass: 353.16	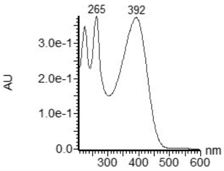	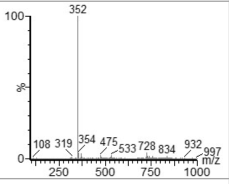	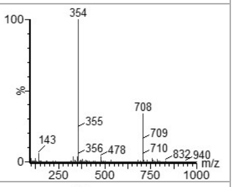
**7**	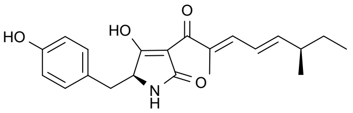 pretenellin A isomer<br>Mass: 355,18	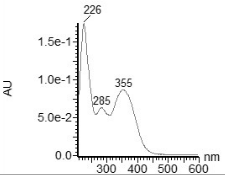	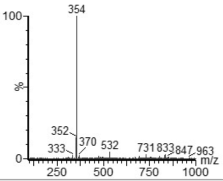	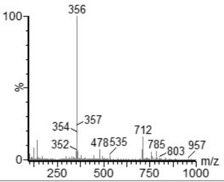
**8**	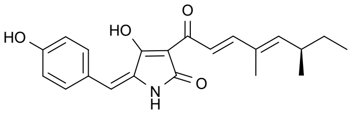 anhydropretenellin A<br>Mass: 353,16	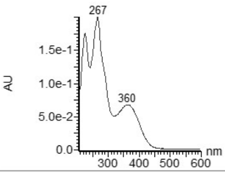	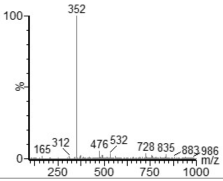	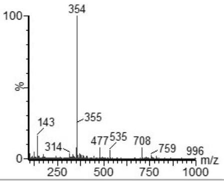
**12**	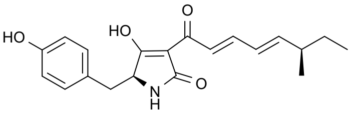 desmethyl pretenellin A<br>Mass: 341.16	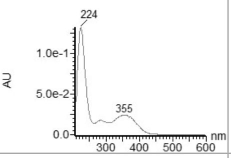	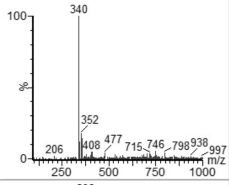	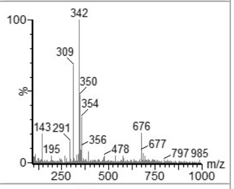
**10**	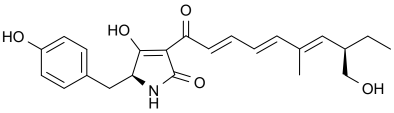 hydroxy prebassianin A<br>Mass: 397.19	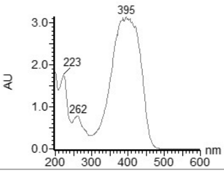	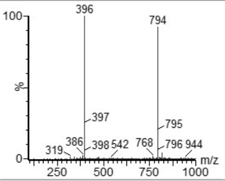	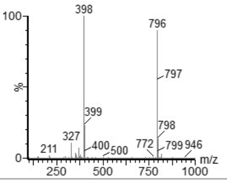
**9**	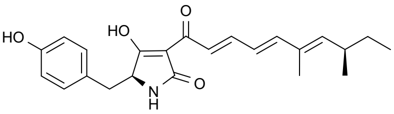 prebassianin A<br>Mass: 381.19	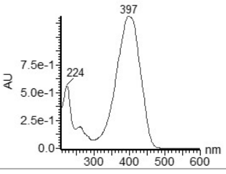	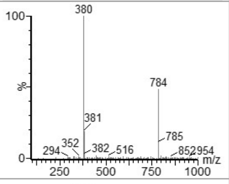	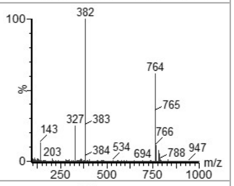
**11**	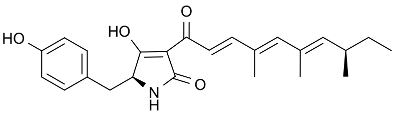 Mass: 395.21	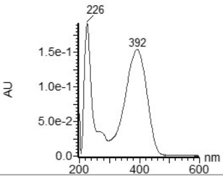	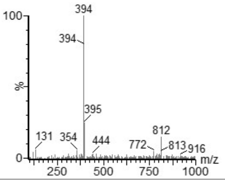	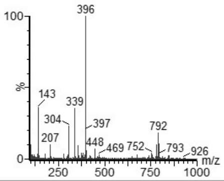

2.2.1 Detailed Analysis of Compound 10

Hexaketide products are produced as a mixture of methylation isomers (see X.-L. Yang, S. Friedrich, S. Yin, O. Piech, K. Williams, T. J. Simpson and R. J. Cox, *Chem. Sci.*, 2019, **10**, 8478–8489).[4] These are hydroxylated to produce a similar mixture of hexaketide alcohols so that the peak indicated as compound 10 actually consists of three isomers. LCMS analysis (below) supports this conclusion.

Expansion of hexaketide alcohol peaks

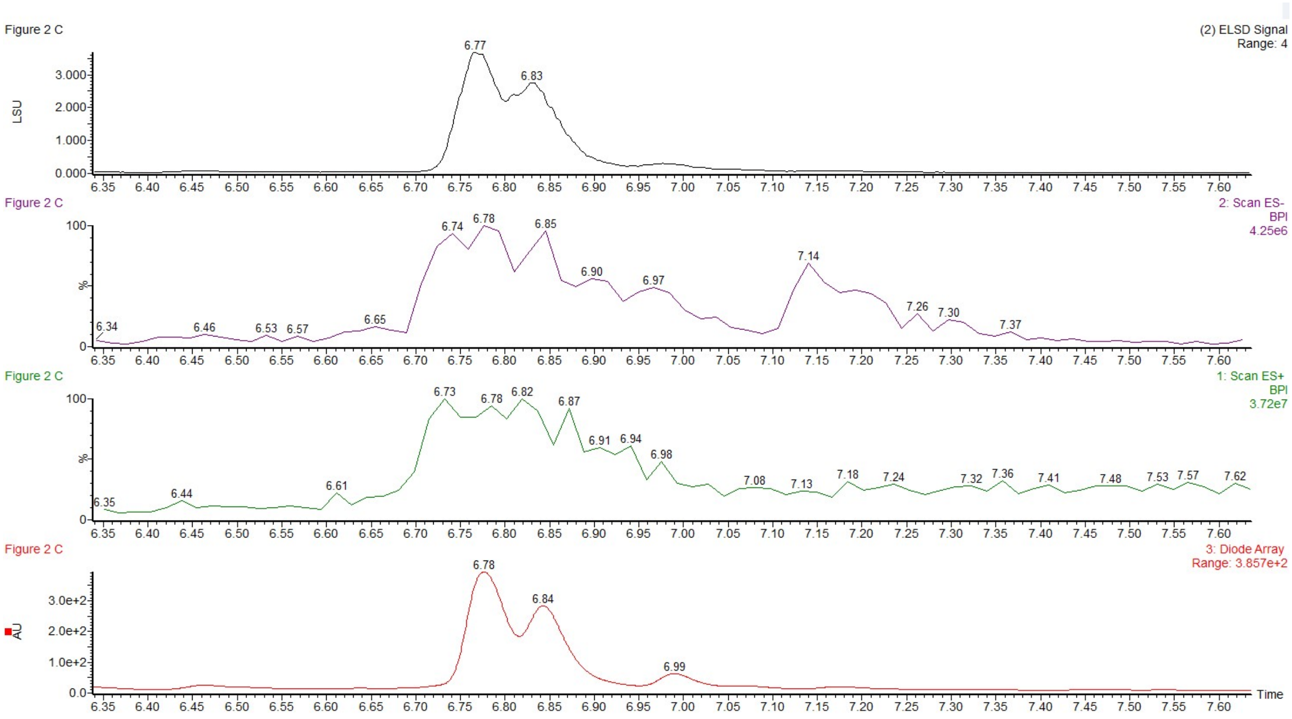

6.78 min peak

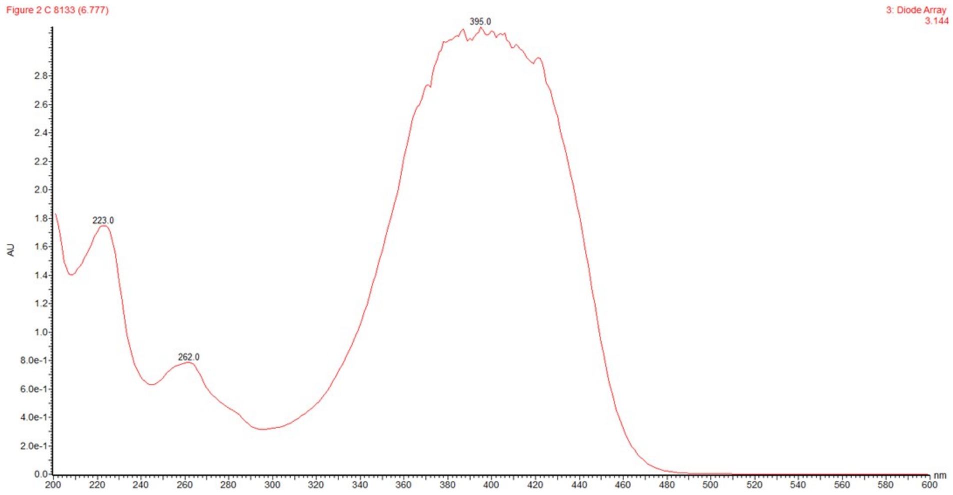
Figure 2 C 8133 (6.777)
3: Diode Array
3.144
395.0
223.0
262.0
AU
nm

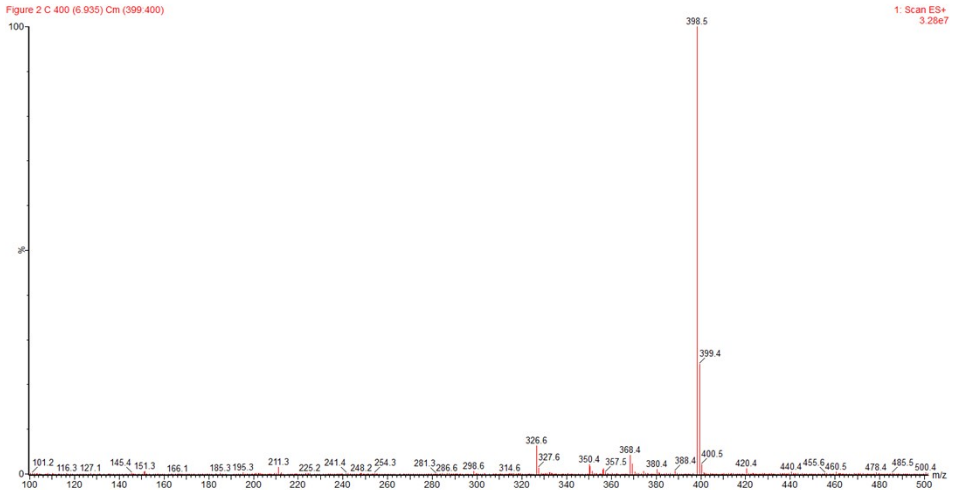
Figure 2 C 400 (6.935) Cm (399:400)
1: Scan ES+
3.28e7
398.5
399.4
326.6
368.4
m/z

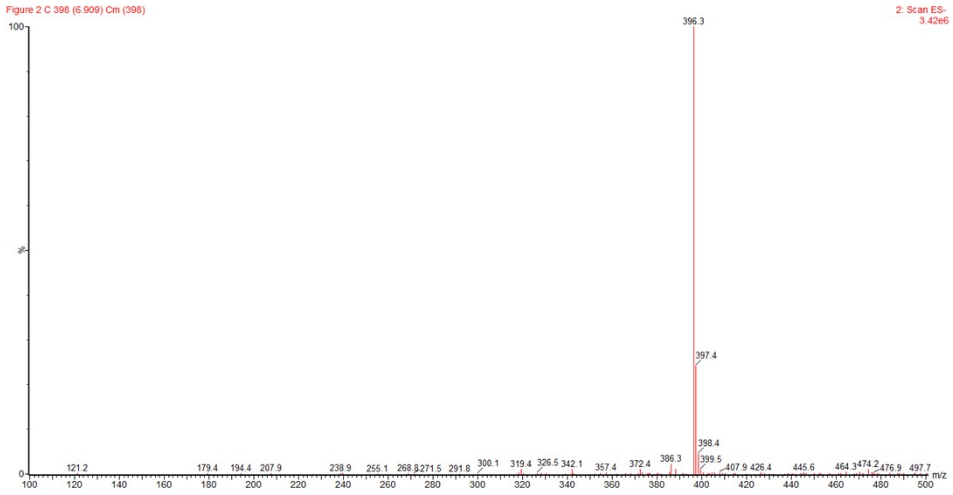
Figure 2 C 398 (6.909) Cm (398)
2: Scan ES-
3.42e6
396.3
397.4
398.4
m/z

6.84 min peak

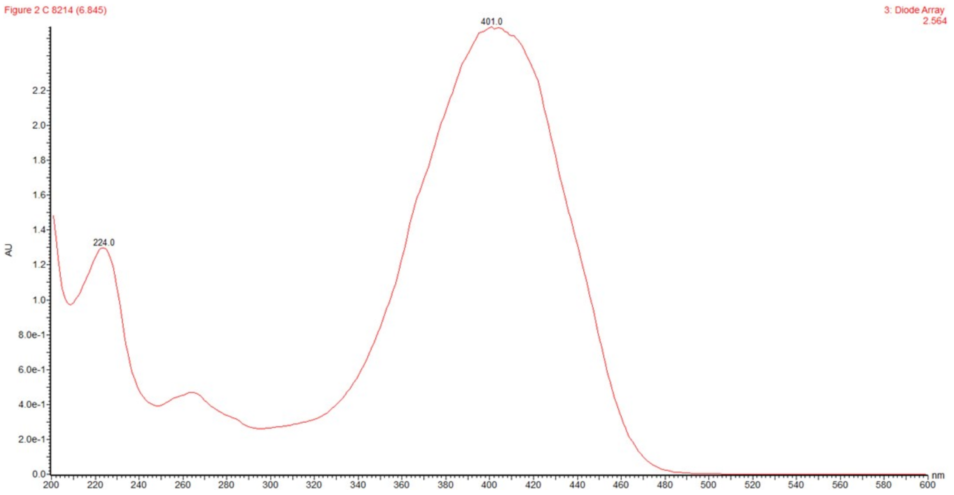
Figure 2 C 8214 (6.845)
3: Diode Array
2.564
401.0
224.0
AU
nm

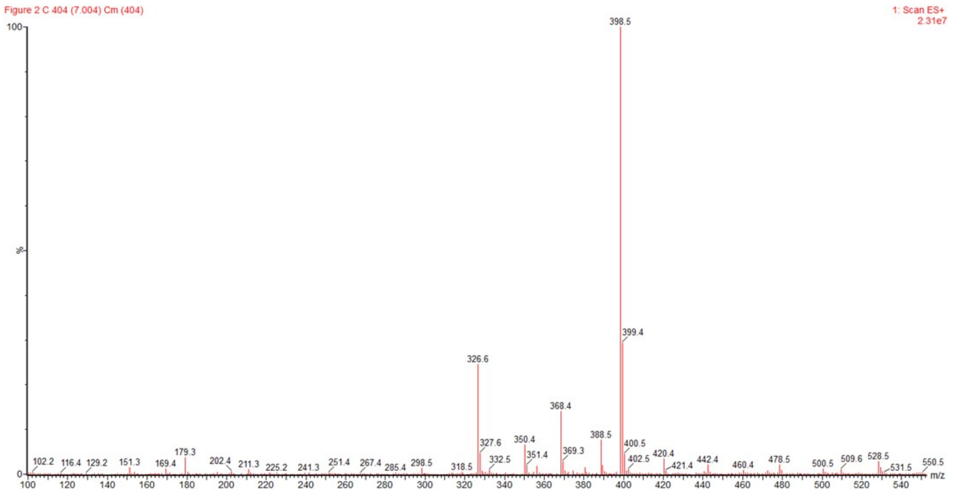
Figure 2 C 404 (7.004) Cm (404)
1: Scan ES+
2.31e7
398.5
399.4
326.6
368.4
388.5
%
m/z

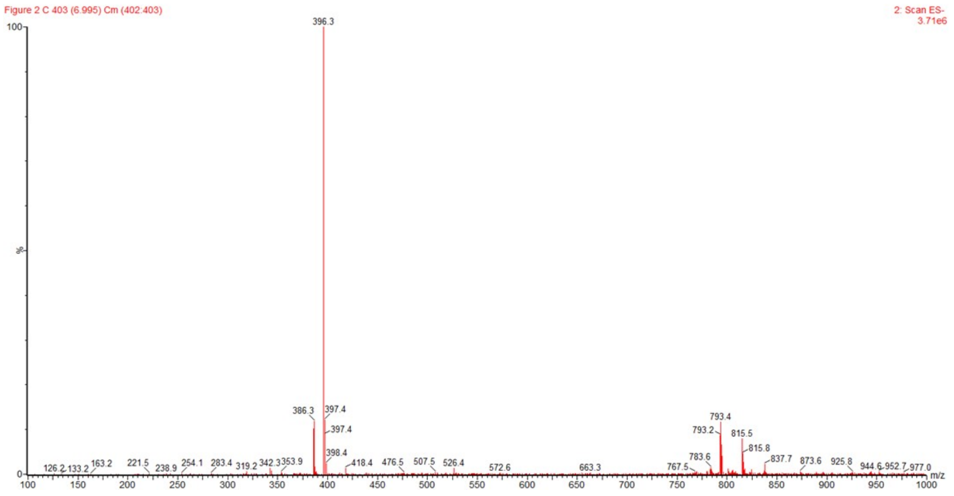
Figure 2 C 403 (6.995) Cm (402:403)
2: Scan ES-
3.71e6
396.3
386.3
397.4
793.4
815.5
%
m/z

6.99 min peak

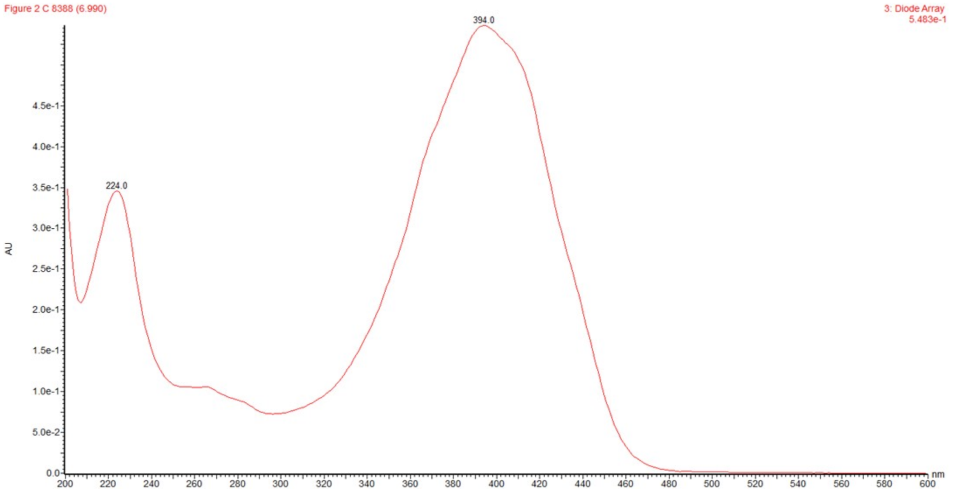
Figure 2 C 8388 (6.990)
3: Diode Array
5.483e-1
394.0
224.0
AU
nm

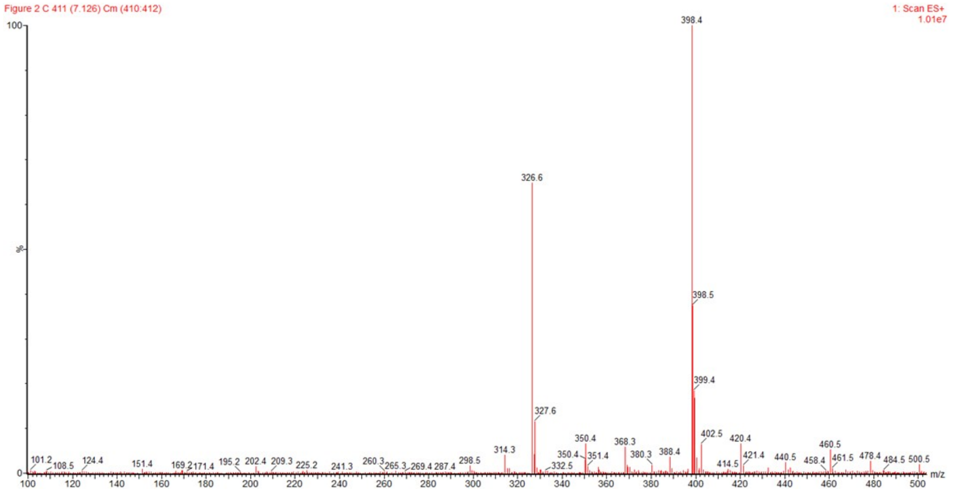
Figure 2 C 411 (7.126) Cm (410:412)
1: Scan ES+
1.01e7
398.4
326.6
398.5
399.4
327.6
%
m/z

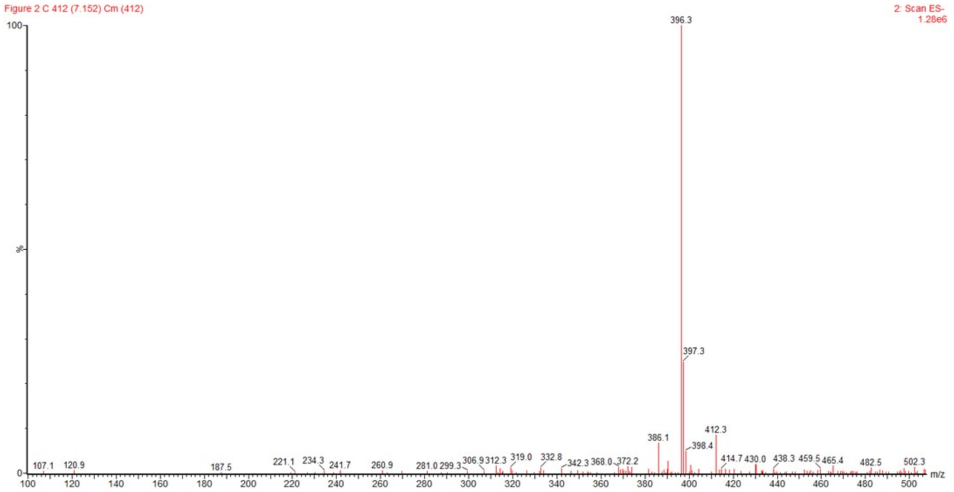
Figure 2 C 412 (7.152) Cm (412)
2: Scan ES-
1.28e6
396.3
397.3
412.3
386.1
398.4
%
m/z

2.3 Overview of all Generated Transformants

Table S7 Overview of all pretenellin A producing transformants.

Experiment	Transformant	Detected compounds						
		Pentaketides				Hexaketides		
		**6**	**12**	**7**	**1**	**9**	**10**	**11**
substrate binding helix = DmbS	A	X		X	X	X	X	
	B	X		X	X	X	X	
	C	X		X	X	X	X	
	D	X		X	X	X	X	
	E			X	X	X	X	
	F	X		X	X	X	X	
	G	X		X	X	X	X	
	H	X		X	X	X	X	
	I	X		X	X	X	X	
substrate binding helix = MilS	A	X		X	X	X	X	X
	B				X	X	X	
	C				X		X	
	D				X	X	X	X
S2400N, L2401R, T2404M, V2406A	A			X	X	X	X	
	B			X	X			
	C			X	X			
	D	X		X	X	X	X	
	E	X		X	X	X	X	
T2395A	A	X		X	X			
	B	X		X	X			
	C	X		X	X			
	D	X	X	X	X			
	E	X		X	X			
	F	X		X	X			
	G	X	X	X	X			
K2396A	A	X	X	X	X			
	B	X	X	X	X			
	C	X		X	X			
	D	X	X	X	X			
	E			X	X			
V2397A	A	X			X			
	B				X			
	C				X			
Q2398A	A	X			X			
	B	X	X		X			
	C	X			X			
	D	X		X	X			
M2399A	A	X		X	X			
S2400A	A	X		X	X			
	B	X		X	X			
	C	X	X	X	X			
	D	X	X	X	X			
	E	X	X	X	X			
L2401A	A		X		X			
	B	X	X		X			
	C				X			

G2402A	A	X		X	X	X		
	B			X	X			
	C	X		X	X	X		
	D	X		X	X	X		
	E	X		X	X	X		
	F	X		X	X			
	G	X		X	X			
	H	X		X	X	X		
	I	X		X	X			
T2403A	A	X	X	X	X			
	B	X	X	X	X			
	C	X	X	X	X			
	D	X	X	X	X			
	E	X	X	X	X			
	F	X	X	X	X			
	G	X	X	X	X			
	H	X	X	X	X			
T2404A	A	X		X	X			
	B	X		X	X			
	C	X		X	X			
	D			X	X			
	E			X	X			
	F							
	G	X		X	X		X	
R2405A	A		X	X	X			
	B	X	X	X	X			
	C	X	X	X	X			
V2406	A	X		X	X		X	
	B	X		X	X		X	
	C	X		X	X	X	X	
M2407A	A			X	X			
	B			X	X			
	C			X	X			
	D	X		X	X			
	E	X	X	X	X	X		X
S2408A	A	X		X	X			
	B	X	X	X	X			
	C	X		X	X			
	D	X	X	X	X			
	E	X	X	X	X			
V2409A	A	X	X	X	X			
	B	X	X	X	X			

3 Multiple Alignment

```
                      2201                                              2250
                      ER     KR                                           KR
TENS         (2196)   PPLQTRGLFKSDRTYLMVGAAGGLGTSICRWMVRNGARHVVVTSRN--PK
DMBS         (2191)   PPLQTRGLFKSDRTYLMVGAAGGLGTSLCRWMVRNGARHVVVTSRN--PK
MILS         (2196)          LFQSDRTYLMVGAAGGVGTSLCRWMVRHGARHVIVTSRN--PK
AmphB        (235)    -------RPPVHGSVLVTGGTGGIGGRVARRLAEQGAAHLVLTSRRGAD-
mFAS pig     (1873)   LTGLSKTFCPPHKSYVITGGLGGFGLQLAQWLRLRGAQKLVLTSRSGIRT
mFAS rat     (1867)   ISAISKTFCPEHKSYIITGGLGGFGLELARWLVLRGAQRLVLTSRSGIRT

                      2251                                              2300
                      KR                                                  KR
TENS         (2244)   ADPEMLNEAERYGAAVQVVPMDACSKDSVQTVVDMIRATMPPIAGVCNAA
DMBS         (2239)   ADPEMLNEAERYGAIVRVVPMDACNKDSVQTVVDTIRATMPPIAGVCNAA
MILS         (2237)   GDPTMLSEAKQYGATVRVVSMDVCDRRSVEAVVGMIRATMPPIACVCNAA
AmphB        (279)    GAAELRAELEQLGVRVTIAACDAADREALAALLAEL-PEDAPLTAVFHSA

mFAS pig     (1923)   GYQARQVREWRRQGVQVLVSTSNASSLDGARSLITEATQLGPVGGVFNLA
mFAS rat     (1917)   GYQAKHVREWRRQGIHVLVSTSNVSSLEGARALIAEATKLGPVGGVFNLA

                      2301                                              2350
                      KR                                                  KR
TENS         (2294)   MVLRDKLFLDMNVDHMKDVLGPKMQGTEHLDSIFAQEP--LDFFVLLSSS
DMBS         (2289)   MVLCDKLFLDMDVDQMNNTLGPKVDGTEYLDSIFAHEP--LDFFILLGSA
MILS         (2287)   MVLCDKLFLDMDVDILNNTLGPKVDGTEILDSIFSEEA--LDFFILLGST
AmphB        (328)    GVAHDD-PVDLTLGQLDALMRAKLTAARHLHELTADL--DLDAFVLFSSG
mFAS pig     (1973)   MVLRDAVLENQTPEFFQDVSKPKYSGTANLDRVTREACPELDYFVIFSSV
mFAS rat     (1967)   MVLRDAMLENQTPELFQDVNKPKYNGTLNLDRATREACPELDYFVAFSSV

                      2351                                              2400
                      KR                                                  KR
TENS         (2342)   AAILNNTGQSNYHCANLYMDSLVTNRRSRGLAASIIHVGHVCDTGYVARL
DMBS         (2337)   AAILNNMGQSNYHCANLYMDSLVKHRRSRGLAASIIHIGHVCDTGYVARM
MILS         (2235)   ATIANNIGQSNYHCANLYMDSLVAQRRSRGLAASIIHIGYICDTGYVARL
AmphB                 AAVFGSGGQPGYAAANAYLDALAEHRRSLGLTASSVAWGTWGEVGMATDP
mFAS pig     (2023)   SCGRGNAGQANYGFANSAMERICEKRRHDGLPGLAVQWGAIGDVGVVLET
mFAS rat     (2017)   SCGRGNAGQSNYGFANSTMERICEQRRHDGLPGLAVQWGAIGDVGIILEA

                      2401                                              2450
                      KR                                                  KR
TENS         (2392)   VDDTKVQMSLGTTRVMSVSETDVHHAFAEAVRGGQPDSRSGSHNIIMGIE
DMBS         (2387)   VDDNRIQSNIATMRAMRLSETDVHHAFAQAVRGGQLDSRSGSYNIIMGIE
MILS         (2385)   GDDAKVHSNRDVMRATTLSETDVHHAFAEAVRGGSPGSPIGSYNIIMGID
AmphB        (337)    EVHDRLVRQGVLAMEPPEHALGALDQMLNDDTAAAPITMDWEMFAPAFTN
mFAS pig     (2073)   MGTNDTVIGGTLPQRIASCLEVLDLFLSQPHPVLS---------------
mFAS rat     (2067)   MGTNDTVVGGTLPQRISSCMEVLDLFLNQPHAVLS---------------
                      2451                                              2500

                      KR                                      KR
TENS         (2442)   PPTKPLDLTKRKPVWISDPRLGPCLPFSTLENQMMASEQAAAASAVDSLA
DMBS         (2437)   PPTKPLDLTRRQAVWLSDPRLGHMLPYSTLENQMIASGQAAA-S-ADSLA
MILS         (2435)   PPTKSLDSSRRKALWLSDPRLGHMVPYSASADQAVTSEQA
AmphB        (478)    RPSALLSTVPEAVSALSDE---------------------
mFAS pig     (2108)   --------------------------SFVLAEKKAAPRDGSSQK------
mFAS rat     (2102)   --------------------------SFVLVEKKAVAHGDGEAQR------
```

: Identity within PKS-NRPS, : Identity within mFAS, RED: Identity between PKS-NRPS and mFAS

4 References

1 R. D. Gietz and R. H. Schiestl, *Nat. Protoc.*, 2007, **2**, 35–37.

2 R. D. Gietz and R. A. Woods, *Methods Mol. Biol.*, 2006, **313**, 107–120.

3 F. Katzen, *Expert Opin. Drug Discov.*, 2007, **2**, 571–589.

4 X.-L. Yang, S. Friedrich, S. Yin, O. Piech, K. Williams, T. J. Simpson and R. J. Cox, *Chem. Sci.*, 2019, **10**, 8478–8489.
